# Epidural abscess after multiple lumbar punctures for labour epidural catheter placement

**DOI:** 10.1016/S1674-8301(10)60046-2

**Published:** 2010-07

**Authors:** Sundeep S. Tumber, Hong Liu

**Affiliations:** Department of Anaesthesiology and Pain Medicine, University of California, Davis, California 95817, USA

**Keywords:** labour, epidural, abscess, catheters, analgesia

## Abstract

Epidural catheterization is routinely used by anaesthesiologists to provide labour and post-operative analgesia. In most cases, catheter placement is without serious side effects and uneventful. However, epidural abscess is a rare complication that may result in severe morbidity. We present a case of epidural abscess after labour epidural catheter placement in a healthy 36-year-old female who presented on post-partum d 10 with complaints of fever and back pain. She was treated with intravenous antibiotics and fully recovered.

## CASE REPORT

The patient, a 36-year-old, gravidity-two parity-one (G2P1) female presented to the hospital at 37 w of gestation for induction of labor secondary to intrauterine growth retardation and increased blood pressure. Her past medical history included hypertension. The patient had no known drug allergy, and she denied any illicit drug use. The only medication the patient took was a prenatal vitamin. Her weight was 72 kg and height was 158 cm. A review of systems, vital signs, laboratories and physical exam was unremarkable. The patient was evaluated by the anesthesia team for possible labor epidural placement before induction of labor.

Induction was started with oxytocin shortly after the completion of evaluation, contractions were regular, and the cervix was dilated to 4 cm within 60 min. The patient desired to have epidural analgesia for labor pain at that time and consent was obtained. Hands were washed with a cholorhexidine soap solution and a mask, cap, and sterile gloves were worn by the anesthesiologist. The patient was placed in the sitting position and the skin of the lumbar region was prepared with povidone iodine with three separate sponges from the epidural kit (B. Braun Medical Inc., USA). Five mililiter 1% lidocaine were obtained from the epidural kit and used for local anesthesia. Two attempts were made with a 17 gauge Tuhoy epidural needle at L3-L4 level and then at the level of L2-L3 without success due to contact with bone. Three milliliter 1% lidocaine was then injected subcutaneously at the L4-L5 interspace and the 17 gauge Tuhoy needle was advanced without difficulty and loss of resistance (LOR) to air was obtained. The epidural catheter was advanced without difficulty and a test dose of 3 mL of 1.5% lidocaine with 1:200,000 of epinephrine was negative. A sterile occlusive dressing was placed over the entry site and the epidural catheter was secured. A bolus of 0.125% bupivicaine 10 mL with 100 mcg of fentanyl was given through a 0.22 µm filter. An infusion of bupivicaine 0.1% with 2 mcg/mL fentanyl was prepared by the hospital pharmacy and started at 10 mL/h. The delivery was completed approximately 45 min after epidural catheter placement. Because the patient requested a procedure of post-partum tubal ligation, the epidural catheter was left in place to be used for the procedure. The next day the patient was taken to the operating room for the tubal ligation procedure. At that time, the epidural catheter was occluded and was therefore removed. The epidural catheter had been in place for 28 h. Spinal anesthesia *via* a 25 gauge Sprotte needle was placed at L4-L5 without difficulty using sterile technique. For skin preparation, a 10% povidone iodine aerosol spray (Cardinal Health, Inc., USA) was used. A mask and sterile gloves were worn by the anesthesiologist. The patient was discharged the next day in stable condition without complaints.

**Fig. 1:**
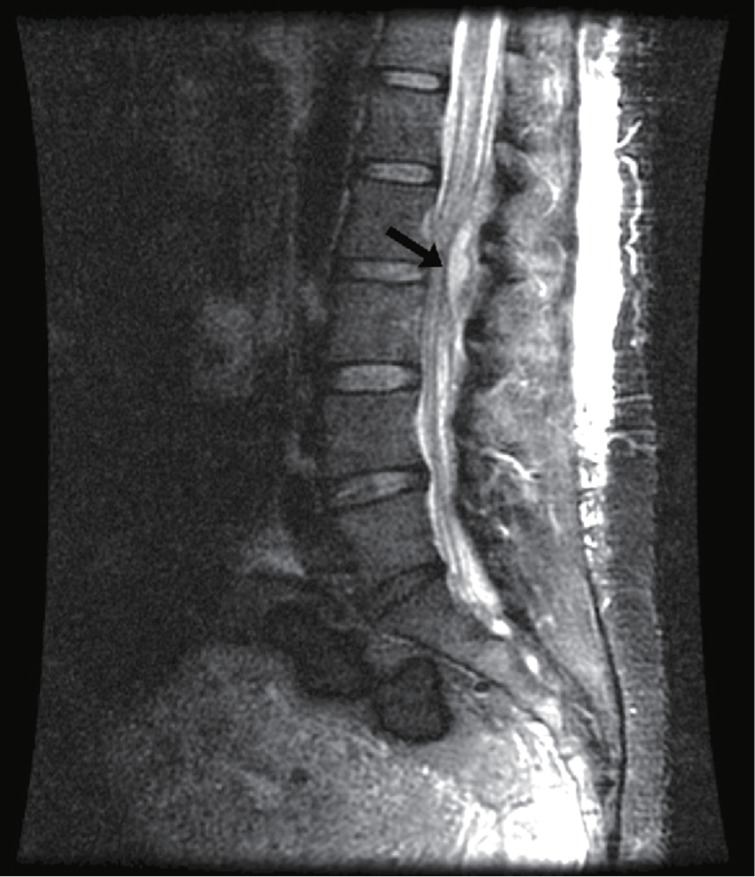
T2 weighted gadolinium MRI image of the lumbar spine. Abnormal enhancement is shows most notably at L2-L3 with 50% cord compression due to a posterior enhancing fluid collection in the epidural space (arrow).

The patient returned to the hospital on post-partum day 10 with complaints of fever (39.0°C), chills, and severe back pain radiating to her lower extremities. She denied bowel/bladder incontinence, loss of sensation, or photophobia, but she did complain of difficulty standing secondary to pain. On physical examination, the epidural insertion site revealed tenderness at the lumbar region without erythema or discharge. Neurological examination revealed intact reflexes and sensation with negative Kernig's and Brudzinksi's signs. Laboratory results revealed a normal white blood cell count of 9.8×10^3^/mm^3^. Blood cultures were taken at this time. A neurosurgery consult was obtained and a magnetic resonance image (MRI) examination was ordered. MRI revealed abnormal enhancement most notably at L2-L3 with 50% cord compression due to a posterior enhancing fluid collection in the epidural space. These findings were consistent with epidural abscess (***Fig. 1***).

CT scan guided drainage for cultures were performed and antibiotic therapy was commenced. As the patient had no neurological deficits, surgical intervention was not indicated. Both blood cultures and aspirate samples grew penicillin sensitive *Staphylococcus aureus (S. aureus)*. The patient was treated in the hospital for approximately 5 d. She was discharged from the hospital with a peripherally inserted central catheter and received nafcillin for another 6 w. The patient made a full recovery.

## DISCUSSION

Epidural abscess is a rare condition that can cause severe neurological damage if left untreated[1],[2]. The overall incidence rate in the general population is 0.2 to 2.8/(10,000·y) with the peak incidence in people whose ages range between 60 and 70. Risk factors include immunocompromised states, diabetes mellitus, alcoholism, cancer, intravenous drug use, spinal trauma, spinal procedure or surgery[2]. No predisposing condition can be found in 20 percent of patients[4]. Epidural abscesses have been reported in association with epidural anesthesia and chronic indwelling epidural catheters used for pain management (incidence 1 in 1,700 d of catheter use)[3],[5],[6]. The estimated incidence following spinal/epidural anesthesia in obstetric patients is 1 in 500,000 and in all patients overall (including obstetrics) is 1 in 60,000[7]–[10]. In a recent survey of a total of 707,455 cases of central neuraxial block, the authors found 52 cases with major complications[1]. Epidural catheters were placed in 293,050 (41%) patients, and there were 20 complications with the incidence being much lower in the obstetric population[1].

Infection of the epidural space may occur at the time of insertion, or by subsequent contamination from the skin site and spread along catheter track, hematological spread, or by a contaminated syringe or local anesthetic solution[7],[11]–[14]. The most common causative organism is Staphylococcus aureus, followed by Staphylococcus epidermis[12].

Signs and symptoms are nonspecific and can range from low back pain to sepsis, and a delay in treatment can result in irreversible neurological damage or death. Fever and back pain are the most common early symptoms, and local tenderness, with or without neurological deficit, is a usual physical finding. Leukocytosis may be the only abnormal laboratory finding. As the disease progresses, bladder/bowel dysfunction, sepsis, and mental status changes can be seen[2]. It has been reported that in cases of epidural abscess following epidural catheter placement, the median duration of catheterization was 4 d, and the median time to onset of clinical symptoms was 8 d after catheterization[9],[19]. A neurosurgery consult should be obtained once diagnosis is suspected. Blood cultures are positive in 60% of patients and cultures from the epidural site should be obtained by CT-guided or open biopsy. Gadolinium-enhanced MRI is the imaging choice for diagnosis of epidural abscess with a sensitivity of 91%[2],[20],[21].

The treatment of choice in most patients is surgical decompression followed by 4 to 6 w of antibiotic therapy. Nonsurgical treatment may be appropriate in patients with minimal neurological symptoms[2],[22]–[25].

Factors which the anesthesia provider can control to reduce the risk of contamination during epidural catheter insertion include the use of a strict aseptic technique which includes wearing sterile gloves, cap, and a mask[7]. The use of a mask when performing an epidural placement is important because infectious organisms have been isolated from nasal swabs taken from the person who performed the procedure and did not wear a mask[7],[11]. The use of a porous dressing might be less likely to encourage bacterial growth by reduction of the potential for fluid accumulation, but might increase the possibility of secondary pathogen colonization by passage of bacteria through the dressing[7]. The length of time that iodine is left on the skin can be of importance. *In vitro* studies have shown that exposing a suspension of methicillin-resistant *S. aureus* and methicillin-sensitive *S. aureus* to 10% povidone iodine for either 15 or 60 s will result in a 55.2% and 97.7% reduction in mean colony count respectively[7]. Cholorhexidine may be a better choice than iodine. In the same study no organism was cultured after the same suspension was exposed to 0.5% chlorhexidine in 80% ethanol for 15 s[26]. Despite skin sterilization, colonies of gram-positive cocci residing in hair follicles and sweat glands may survive adequate contact with the disinfectant. In addition, the actual disinfectant containers themselves may become colonized by bacteria after previous opening. As a result, single-use packets of disinfectant are now recommended for routine use[27]–[29]. Contamination of epidural solution is a further possible mechanism of infection. Using a closed delivery system and solution prepared by the pharmacy reduces the likelihood of contamination[7]. Use of a 0.22 µm bacterial filter to inject solutions may further reduce the contamination risk[7],[16]–[18].

The sterility of the epidural needle is critical in preventing the introduction of bacteria with the passing of the epidural needle. Epidural needle contamination could be as high as 25% and multiple entries could theoretically results in a higher rate of infection[30]. However, there is no evidence that multiple attempts could cause an epidural abscess[30],[31]. One study with multiple skin passes with epidural needles showed no increased epidural infection up to 5 d post-procedure follow-up[30]. Certainly, the local tissue injury caused by the multiple epidural needle attempts did not help in preventing this complication.

In summary, epidural abscess is a rare but potentially devastating complication of epidural catheter insertion. The signs and symptoms of epidural abscess are nonspecific, and prompt diagnosis and treatment is necessary to prevent permanent neurological injury and death. By presenting this case report, we hope to stress the importance of using sterile technique during placement of epidural catheters and to increase awareness of the presenting signs, diagnosis, and treatment of epidural abscess. Although no evidence implicates multiple attempts with the occurrence of epidural abscess, avoiding multiple attempts could potentially be beneficial.
